# Supplementary material for: Rapid surge of reassortant A(H1N1) influenza viruses in Danish swine and their zoonotic potential
Source: Emerg Microbes Infect. 2025 Feb 13;14(1):2466686. doi: 10.1080/22221751.2025.2466686 (PMC11849018; doi:10.1080/22221751.2025.2466686)
Supplement: Supplementary File_1_clean.docx [file TEMI_A_2466686_SM3641.docx]

**Supplementary File 1.** Nucleotide identity (%) between the segments of the inoculum strains selected for the ferret study (A/swine/Denmark/19922-5/2021 and A/swine/Denmark/15063-1/2020) and the zoonotic H1pdm09N1av (A/Denmark/36/2021).

|  | A/Swine/Denmark/19922-5/2021 and A/Denmark/36/2021 | A/Swine/Denmark/15063-1/2020 and A/Denmark/36/2021 |
| --- | --- | --- |
| PB2 | 99.39% (14 nts) | 93.55% (147 nts) |
| PB1 | 99.65% (8 nts) | 94.46% (126 nts) |
| PA | 99.77% (5 nts) | 94.24% (124 nts) |
| HA | 99.00% (17 nts) | 97.88% (36 nts) |
| NP | 99.74% (4 nts) | 94.79% (79 nts) |
| NA | 99.36% (9 nts) | 98.58% (20 nts) |
| M | 99.58% (4 nts) | 96.20% (36 nts) |
| NS | 99.88% (1 nts) | 79.71% (170 nts) |

The number of nucleotide (nt) differences are indicated in brackets.
