# Supplementary material for: Rapid surge of reassortant A(H1N1) influenza viruses in Danish swine and their zoonotic potential
Source: Emerg Microbes Infect. 2025 Feb 13;14(1):2466686. doi: 10.1080/22221751.2025.2466686 (PMC11849018; doi:10.1080/22221751.2025.2466686)
Supplement: Supplementary File 7.docx [file TEMI_A_2466686_SM3636.docx]

**Supplementary File 7.** Overview of the different length and origin of the NS1 protein identified in the H1N1pdm09 and H1pdm09N1av genotypes

|  | NS1 length  (aa) | H1N1pdm09 Genotype 1  PPPPPP | H1N1pdm09 Genotype 2  PPPPPA | H1pdm09N1av Genotype 1  PPPPPP | H1pdm09N1av Genotype 2  PPPPPA |
| --- | --- | --- | --- | --- | --- |
| *Pdm* | 219 | x |  | x |  |
| *H1N1av* | 230 |  |  |  | x |
|  | 217 |  | x |  |  |

“Pdm” indicates a NS1 origin of H1N1pdm09 whereas “H1N1av” indicates NS1 of H1N1av origin. Aa = indicates Amino acids. There are few exceptions to this generalization of the distribution of the NS1 protein, which is indicated in the phylogenetic tree in Figure 3.
